# Preliminary results of SARS-CoV-2 detection in sewerage system in Niterói municipality, Rio de Janeiro, Brazil

**DOI:** 10.1590/0074-02760200196

**Published:** 2020-07-27

**Authors:** Tatiana Prado, Tulio Machado Fumian, Camille Ferreira Mannarino, Adriana Gonçalves Maranhão, Marilda Mendonça Siqueira, Marize Pereira Miagostovich

**Affiliations:** 1 Fundação Oswaldo Cruz-Fiocruz, Instituto Oswaldo Cruz, Laboratório de Virologia Comparada e Ambiental, Rio de Janeiro, RJ, Brasil; 2 Fundação Oswaldo Cruz-Fiocruz, Escola Nacional de Saúde Pública Sérgio Arouca, Departamento de Saneamento e Saúde Ambiental, Rio de Janeiro, RJ, Brasil; 3 Fundação Oswaldo Cruz-Fiocruz, Instituto Oswaldo Cruz, Laboratório de Vírus Respiratórios e do Sarampo, Rio de Janeiro, RJ, Brasil

**Keywords:** SARS-CoV-2, sewage, Niterói

## Abstract

This study presents preliminary results from a sewage-based surveillance to monitor the spread of severe acute respiratory syndrome coronavirus 2 (SARS-CoV-2) in the municipality of Niterói, State of Rio de Janeiro, Brazil. By using ultracentrifugation method associated to quantitative reverse transcription polymerase chain reaction (RT-qPCR) we detected SARS-CoV-2 in 41.6% (5/12) of raw sewage samples obtained from sewage treatment plants and sewers network in the city. This pioneer study carried out in Brazil aims to subsidise information for health surveillance concerning the viral circulation in different areas of the city and, revealed the insertion and importance of environmental virology in health public policies.

Similar to many other viruses-causing respiratory syndromes, the main transmission route of severe acute respiratory syndrome coronavirus 2 (SARS-CoV-2) is through respiratory droplets generated by coughing and/or sneezing, although the route of contamination by fomites is also considered.^1^ However, diarrhoea has been described in a significant number of cases (incidences varying from 2% to 50% of cases), with viral loads ranging up to 1 x 10^6^ genome copies per g of faecal material.^2^ ^,^ ^3^ Consequently, the presence of SARS-CoV-2 has been also detected in sewage samples from different countries, such as the Netherlands, Australia, France and USA.^4^ ^,^ ^5^ ^,^ ^6^ ^,^ ^7^ These studies have demonstrated the importance of sewage-based surveillance, for an early detection of new cases, as recently found in the shedding/excretion of coronavirus by infected people two to three days before the onset of symptoms.^8^

In this context, a pilot project was underway to investigate the dissemination of SARS-CoV-2 in sewage system in areas of interest for health surveillance in the city of Niterói, in order to monitor the transmission pattern over the period of the epidemic. Niterói notified the first Coronavirus Disease 2019 (COVID-19) case on 12th March 2020, and by now (April 24), seventeen fatal cases associated with COVID-19 were reported.^9^ Until April, 2020 (17th epidemiological week), the city had 254 confirmed cases of SARS-CoV-2, distributed in 32 of its 52 neighborhoods.^9^ The maximum lethality rate was of 14.29%, in the second week after the beginning of the registered cases, decreasing to 6.69% in the 15th epidemiological week.^9^

On April 15th, raw sewage samples were obtained at 12 different sampling points in the city of Niteroi, including sewage treatment plants (STPs), hospital wastewater and sewers network (Figure). Ten-hour composite sewage samples were collected in sterile polypropylene bottles and pasteurised at 60ºC for 90 min to inactivate the virus.^6^ The concentration of the viral particles was performed using ultracentrifugation method, as previously described.^10^ For detection of SARS-CoV-2, 140 µL of suspended viral concentrates were extracted using the QIAamp^®^ Viral RNA Mini kit (QIAGEN, CA, USA) and a QIAcube^®^ automated system (QIAGEN) and coronavirus RNA was detected by quantitative reverse transcription polymerase chain reaction (RT-qPCR). Primers and probe previously published by the Centres for Disease Control and Prevention (CDC) 2020^11^ targeting the N2 region of SARS-CoV-2 genome were used and RT-qPCR was performed in a 15 µL final volume reactions, including 5 µL of template and 3.82 uL of mix contained in the diagnostic kit (Reagentes para Detecção de SARS-CoV2, N1, N2, N3 protocolo CDC - Bio-Manguinhos), according to manufacturer’s instructions (Instituto de Tecnologia em Imunobiológicos - Bio-Manguinhos, Fundação Oswaldo Cruz, Rio de Janeiro, Brazil). Reactions were run according to following conditions: 51ºC at 30 min for reverse transcription, 95ºC at 10 min for denaturation, 45 cycles of 95ºC at 30 s followed by 58ºC at 30 s. The reactions were carried out in duplicate on undiluted and diluted (1:10) RNA samples. Reactions were considered positive for samples showing cycle threshold (Ct) values below 40 cycles recorded for at least two of the four wells tested for each sample.

We detected SARS-CoV-2 RNA in five of 12 (41.6%) samples that presented mean Ct values ranging from 36.3 to 39.8 (Table). All positive samples had at least two positive reactions, from diluted or undiluted samples. The majority of positive samples were detected in those collected from Icaraí neighborhood, and reflects the higher number of reported COVID-19 cases (n = 70) in Niterói until the collection date. We also detected SARS-CoV-2 RNA in one sample at Camboinhas STP, demonstrating the expansion of the outbreak to other areas of the city (Figure). Since there is still no consensus on the use of a set of more effective primers to detect SARS-Cov-2 in environmental samples,^4^ we believe that new studies using primers targeting different genome regions should be conducted to compare the effectiveness of the method.

**TABLE:** Detection of severe acute respiratory syndrome coronavirus 2 (SARS-CoV-2) according to collection sites at Niterói

Collection points	Neighborhood	UTM coordinates (X,Y)^*^	Cycle treshold^**^
P1 - Private hospital	Centre	693559,04257000000 7466942,19405000000	-
P2 - Municipal hospital	Fátima	693803,04180800000 7466756,50910000000	-
P3 - SW^a^	Ingá	692173,23553200000 7465944,85841000000	-
P4 - SW	Icaraí	693909,46129600000 7466382,32137000000	38.7
P5 - SW	Icaraí	694301,25708000000 7465690,99549000000	36.3
P6 - SW	Icaraí	694374,19755900000 7465403,54717000000	-
P7 - STP^b^	Icaraí	694498,85889200000 7465360,03900000000	36.8
P8 - SW	Icaraí	695102,42759900000 7465624,31859000000	38.5
P9 - SW	São Francisco	695264,53813100000 7464630,92966000000	-
P10 - SW	Charitas	694816,88428500000 7462495,56150000000	-
P11 - SW	Jurujuba	693422,85629500000 7462316,67194000000	-
P12 - STP^c^	Camboinhas	699193,05991900000 7460506,52145000000	39.8

a: SW sewers network; b: sewage treatment plant inflow = 1350 L/s; c: STP inflow = 116 L/s; *: SIRGAS 2000 23S; **: cycle threshold is represented by the mean of undiluted samples.

**Figure:**
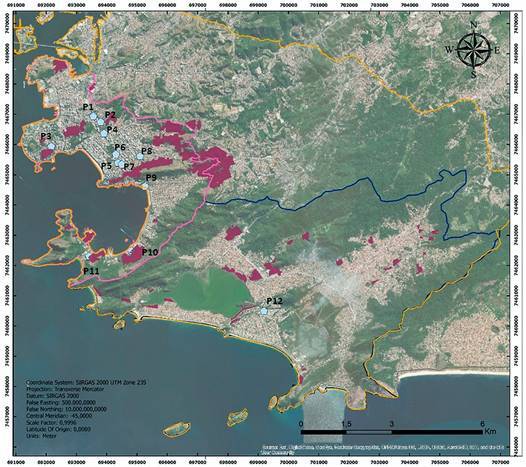
Map of the municipality of Niterói with sampling points. Source: Sistema de Gestão da Geoinformação (SIGEO). Secretaria de Planejamento, Orçamento e Modernização da Gestão. Prefeitura de Niterói.

To the best of our knowledge, there is no direct evidence to prove that the SARS-CoV-2 detected in the sewage system is infectious and contagious. Therefore, it is early to state that wastewater could be considered an important route of transmission. However, with the increase circulation of the virus in the population, the concentration of coronavirus particles in sewage waters could reach significant levels. Thus, it is important to monitor the occurrence and dissemination of SARS-CoV-2 in the sewerage system, identifying COVID-19 hotspots areas, as well as those areas with underreported cases in the health system. Through assessment of viral load and its distribution in sewers network in different areas of the city, the monitoring of the coronavirus circulation during the epidemic will subsidize information for health surveillance, allowing optimising the use of available resources and strengthening measures for prophylaxis in the area. This study confirms wastewater-based surveillance as a promising approach to understand the prevalence of the virus in a given community and the insertion of environmental virology in health public policies.^12^
